# Robotic thyroidectomy versus endoscopic thyroidectomy: a meta-analysis

**DOI:** 10.1186/1477-7819-10-239

**Published:** 2012-11-09

**Authors:** Shuang Lin, Zhi-Heng Chen, Hong-Gang Jiang, Ji-Ren Yu

**Affiliations:** 1 Department of Oncological surgery, First Affiliated Hospital of Jiaxing University, Jiaxing, 314000, Zhejiang Province, China; 2 Department of Surgery, First Affiliated Hospital, Medical College, Zhejiang University, Hangzhou, 310003, Zhejiang Province, China

**Keywords:** Robotic thyroidectomy, Endoscopic thyroidectomy, Da Vinci robotic system, Meta-analysis

## Abstract

**Background:**

To conduct a meta-analysis to determine the relative merits of robotic thyroidectomy (RT) and endoscopic thyroidectomy (ET).

**Methods:**

A literature search was performed to identify comparative studies reporting peri-operative outcomes for RT and ET. Pooled odds ratios (ORs) and weighted mean differences (WMDs) with 95% confidence interval (95% CI) were calculated using either a fixed-effects or a random-effects model.

**Results:**

Six studies matched the selection criteria, which reported on 2048 subjects, of whom 978 underwent RT and 1070 underwent ET. Comparing the outcomes of RT with ET, this meta-analysis indicated that RT was associated with more complications (WMD = 1.51, 95% CI 1.18 to 1.94) and greater amount of drainage fluid (WMD = 17.10, 95% CI 5.69 to 28.51). Meanwhile, operating time (WMD = 1.50, 95% CI −39.59 to 42.58), conversion (WMD = 0.63, 95% CI 0.07 to 6.17), post-operative hospital stay (WMD = −0.05; 95% CI −0.18 to 0.08), and the number of lymph nodes harvested (WMD = 0.62, 95% CI −0.29 to 1.53) were similar for both procedures.

**Conclusion:**

The results of this meta-analysis indicated that RT is associated with an increased risk of complications and a greater amount of drainage fluid. Therefore, RT does not appear to have any advantage over ET. Further studies are required to confirm these results.

## Review

### Introduction

Since the first report of endoscopic thyroid lobectomy in 1997 [1], various endoscopic thyroid techniques or approaches have been described [2,3]. Many studies have reported several advantages of endoscopic thyroidectomy (ET) compared with open thyroidectomy, including better cosmetic results, a lower rate of post-operative complications, and better completion rate for surgery [4,5]. However, ET remains a technically challenging procedure. The two-dimensional visual representation and use of nonflexible endoscopic instruments can make it difficult to visualize the surgical field adequately and to manipulate instruments.

The Da Vinci robotic system was developed to improve the weak points of endoscopic surgery, and surgical robots have been successfully applied to a number of disciplines [6-8]. Recent studies have reported that robotic thyroidectomy (RT) is a feasible, safe, and effective method of performing such surgeries [9,10], although most studies have been limited by small samples size and assessment at a single institution.

In this study, we aimed to determine the relative merits of RT and ET by performing a meta-analysis of studies comparing the two techniques.

## Methods

### Study selection

The Pubmed, EMBASE, Cochrane Library, Ovid, and Web of Science databases were searched systematically for all articles published in English before July 2012 that compared RT and ET. The terms used for the search were: ‘robotic’ and ‘thyroidectomy’.

Reference lists of all retrieved articles were also manually searched for additional studies. Two reviewers independently extracted the data from each study. All relevant text, tables, and figures were reviewed for data extraction. Discrepancies between the two reviewers were resolved by discussion and consensus.

### Inclusion and exclusion criteria

Only studies in the English language were considered for inclusion. In addition, each study had to fulfill the following criteria: 1) it compared the outcomes of RT and ET, and 2) it reported on at least one of the outcome measures mentioned below. In cases where dual (or multiple) studies were reported by the same institution and/or authors, either the higher-quality or the most recent publication was included in the analysis.

Abstracts, letters, editorials and expert opinions, reviews without original data, case reports, and studies lacking control groups were excluded. The studies or data were also excluded when: 1) the outcomes and parameters of patients were not clearly reported (for example, with no clearly reported outcomes or standard deviations (SDs)); 2) it was impossible to extract the appropriate data from the published results; or 3) there was overlap between authors or centers in the published literature.

### Outcomes of interest and data extraction

The following outcomes were used to compare the two operating techniques: 1) intra-operative data, which included operating time (min), and conversion; 2) post-operative data, which included complications, amount of drainage fluid (ml), and post-operative hospital stay (days); and 3) pathologic details, which included number of lymph nodes harvested.

Two reviewers independently extracted the following parameters from each study: 1) first author and year of publication; 2) study population characteristics; 3) number of subjects who underwent each technique; and lastly, 4) intra-operative data, post-operative data, and pathologic details.

### Statistical analysis

The meta-analysis was performed using the Review Manager (RevMan) software, (version 4.2.2; Cochrane IMS; http://ims.cochrane.org). We analyzed dichotomous variables using estimation of odds ratio (OR) with 95% confidence interval (CI) and continuous variables using weighted mean difference (WMD) with 95% CI. The pooled effect was calculated using either a fixed-effects or a random-effects model. Heterogeneity between studies was evaluated using the *χ*^2^ and I^2^ tests, and we considered heterogeneity to be present if the I^2^ statistic was >50%. *P* < 0.05 was considered significant.

## Results

### Selection of trials

**Figure 1:**
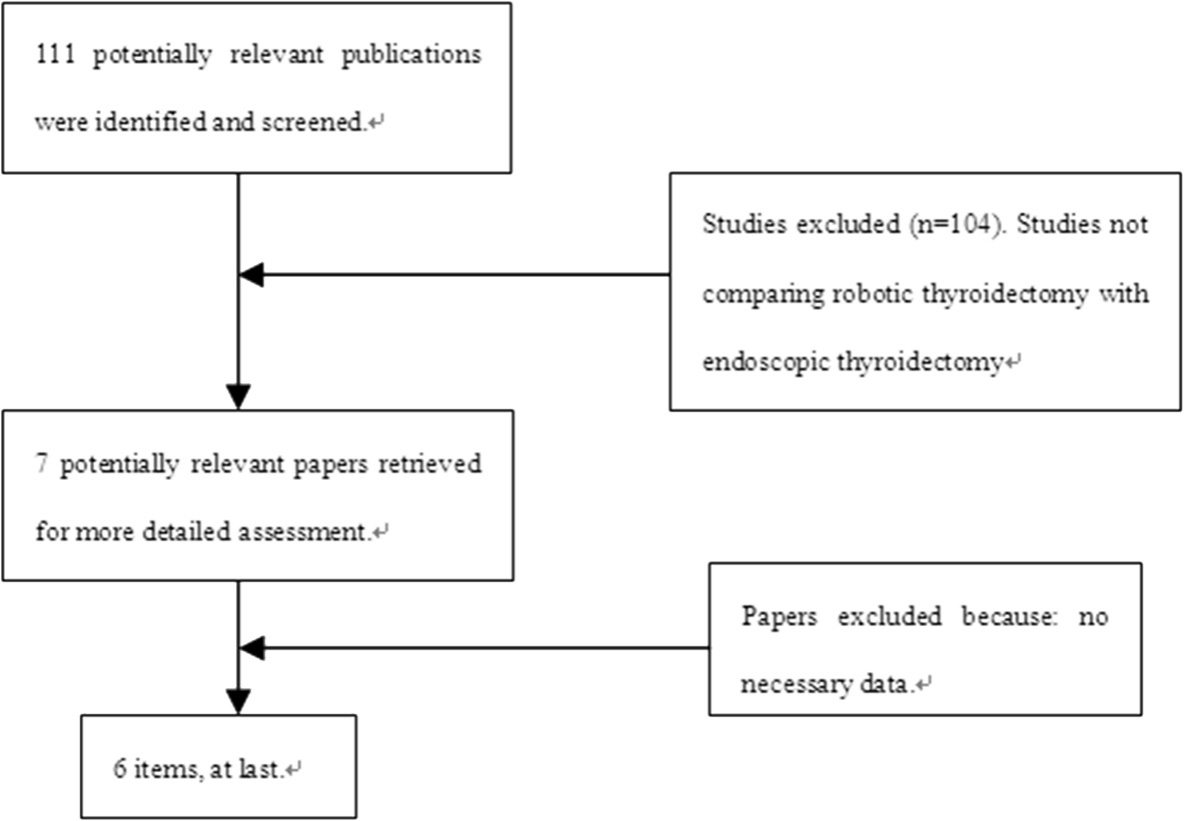
Flow chart for the selection process.

The initial search strategy retrieved 111 publications, after screening all titles, abstracts, and full text. Six studies [11-16] met our entry criteria, and were retrieved for more detailed evaluation (Figure 1). All six studies were non-randomized controlled trials, and their characteristics are summarized in Table 1. The total number of patients in all the trials was 2048. RT was performed on 978 patients and ET was on 1070 patients. The indications and exclusion criteria for surgery varied between the trials, and the post-operative histologic diagnosis are illustrated in Tables 2 and 3.

**Table 1:** Characteristics of included studies

**Study**	**Author**	**Year**	**Design**	**Group**	**Patients in each group, n**
1	Yoo HH *et al*.	2012	NRCT	RT/ET	46/165
2	Tae K *et al*.	2012	NRCT	RT/ET	113/105
3	Lee S *et al*.	2011	NRCT	RT/ET	580/570
4	Lee J *et al*.	2011	NRCT	RT/ET	163/96
5	Lang BHH *et al*.	2011	NRCT	RT/ET	7/39
6	Kim WW *et al*.	2011	NRCT	RT/ET	69/95

ET, endoscopic thyroidectomy; NRCT, nonrandomized controlled trials; RT, robotic thyroidectomy.

**Table 2:** Indication and exclusion for surgery

	**Trial**	**Indication for surgery**	**Exclusion criteria**
1	Yoo HH *et al*.	Benign thyroid nodules	Nodule > 40 mm diameter
		Potentially thyroid nodules	Poorly differentiated thyroid cancer
			Lateral lymph-node metastasis
			Distant metastasis
			Invasion to adjacent organs
2	Tae K *et al*.	Benign thyroid nodules	Nodule > 50 mm diameter
		Follicular neoplasm	Nodule > 50 mm diameter
		Differentiated thyroid carcinoma	Nodule > 10 mm diameter
			Cervical lymph-node metastasis
			No single
3	Lee S *et al*.	Well-differentiated thyroid carcinoma	Multiple lateral cervical lymph-node metastases
			Distant metastasis
			Invasion to adjacent organs
			Location in the thyroid dorsal area
4	Lee J *et al*.	Follicular neoplasm	Nodule > 50 mm diameter
		Differentiated thyroid carcinoma	Nodule > 30 mm diameter
			Multiple lateral cervical lymph- metastases
			Distant metastasis
			Invasion to adjacent organs
			Previous neck surgery
			Severe Graves’ disease
			Location in the thyroid dorsal area
5	Lang BHH *et al*.	Benign thyroid nodules	Nodule > 40 mm diameter
		Potentially thyroid nodules	Nodule > 20 mm diameter
6	Kim WW *et al*.	Papillary thyroid microcarcinoma	Nodule > 10 mm diameter
			Central cervical lymph- node metastases
			Distant metastasis
			Invasion to adjacent organs
			Severe thyroiditis

**Table 3:** Post-operative histologic diagnosis

	**Trial**^**a**^	**Benign**^**b**^	**Malignant**^**b**^
1	Yoo HH *et al*.	2:17	44:148
2	Tae K *et al*.	21:59	92:46
3	Lee S *et al*.	–	580:570
4	Lee J *et al*.	11:41	152:55
5	Lang BHH *et al*.	6:35	1:4
6	Kim WW *et al*.	–	69:95

^a^In these studies, patients in the two groups were matched for operation time [11-14,16], conversion [11-16], complication [11-16], amount of drainage fluid [11,12,14,16], length of post-operative hospital stay [11-14,16], number of lymph nodes harvested [11-14,16].

^b^Benign/malignant: ratio of post-operative histology in robotic thyroidectomy or endoscopic thyroidectomy. Two studies did not have any benign tumors included.

### Meta-analysis of intra-operative data

**Figure 2:**
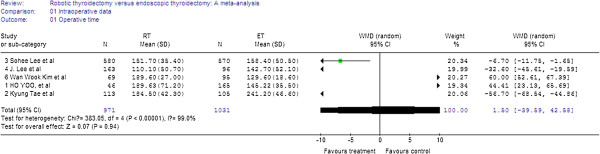
Forest plot displaying the results of the meta-analysis for operating time.

In the five studies, there was no significant difference in the operating time between the RT and the ET groups (WMD = 1.50, 95% CI −39.59 to 42.58). The random effects model was used because of the heterogeneity (I^2^ = 99.0%) (Figure 2).

**Figure 3:**
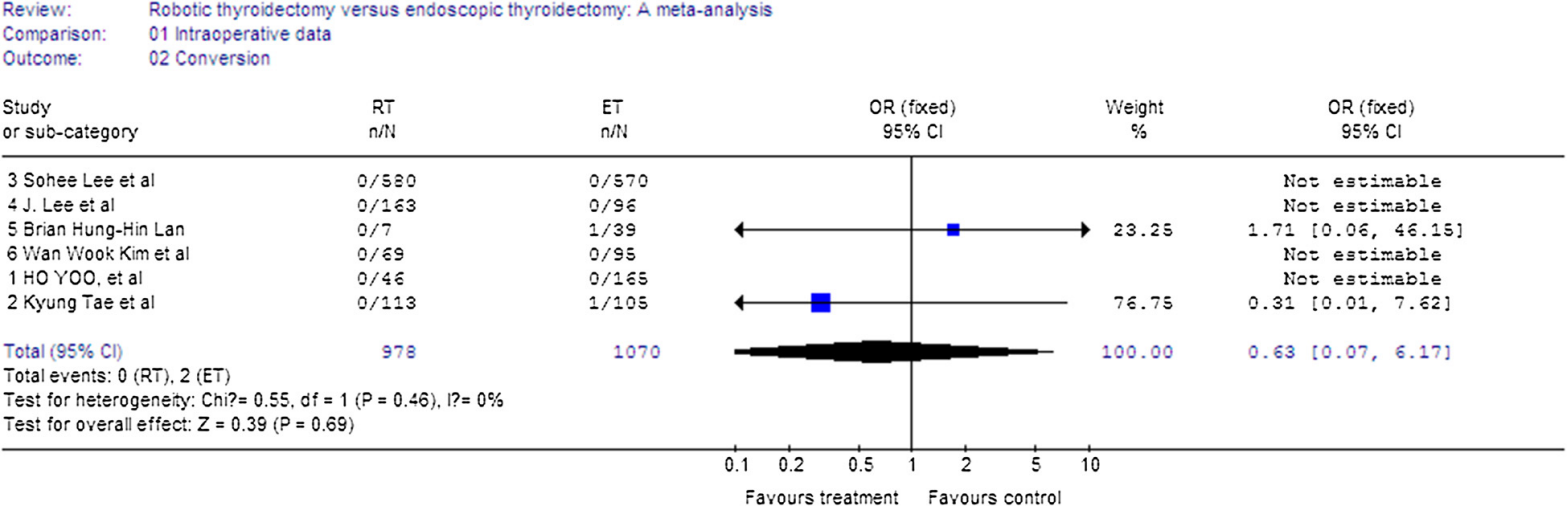
Forest plot displaying the results of the meta-analysis for conversion.

Six studies reported on conversion; there was no significant difference between the groups (WMD = 0.63, 95% CI 0.07 to 6.17). There was no significant heterogeneity between the studies (I^2^ = 0%) (Figure 3).

### Meta-analysis of post-operative outcomes

**Figure 4:**
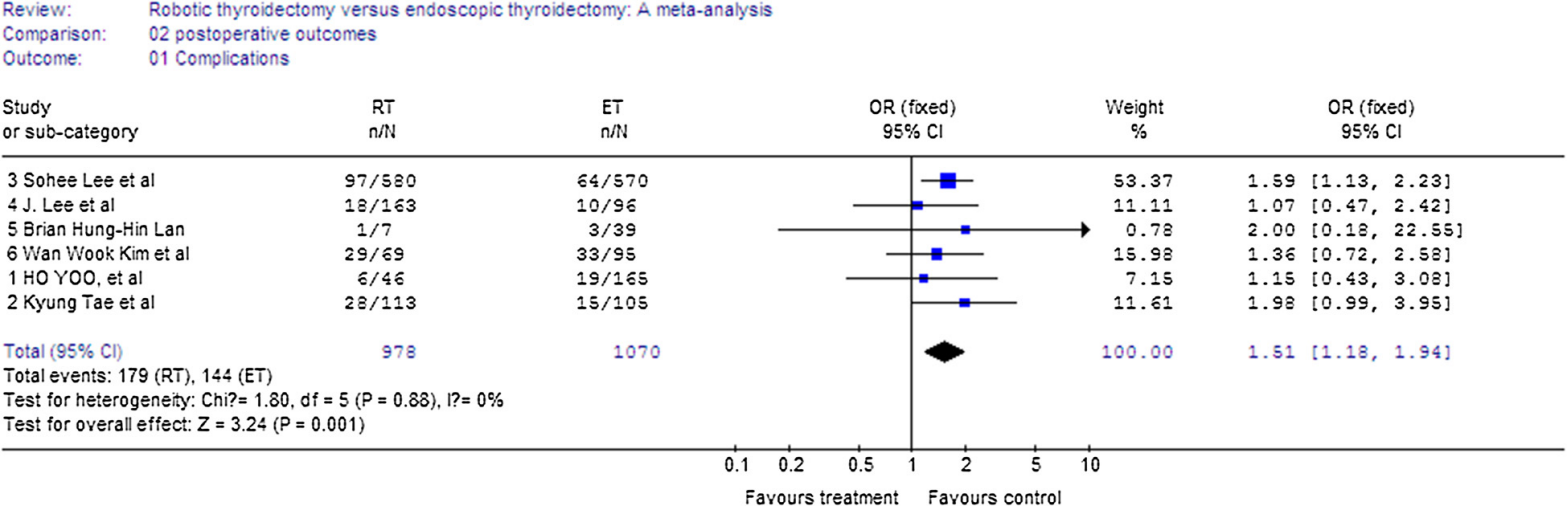
Forest plot displaying the results of the meta-analysis for complications.

In the six studies, there was a significant difference in complications between the RT and the ET groups, with RT found to have more complications (WMD = 1.51, 95% CI 1.18 to 1.94). There was no significant heterogeneity between the studies (I^2^ = 0%) (Figure 4).

**Figure 5:**
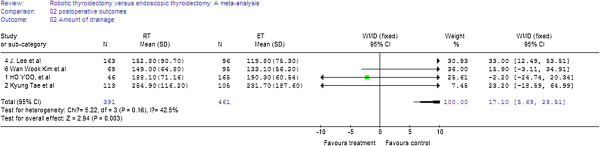
Forest plot displaying the results of the meta-analysis for amount of drainage.

Four studies reported on amount of drainage fluid Analysis of the pooled data showed that patients in the RT group had a greater amount of drainage fluid (WMD = 17.10, 95% CI 5.69 to 28.51) (Figure 5).

**Figure 6:**
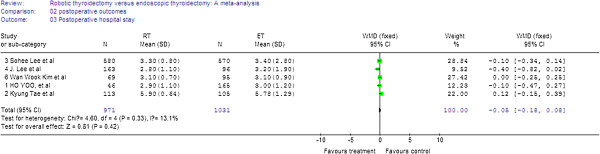
Forest plot displaying the results of the meta-analysis for post-operative hospital stay.

For the five studies, analysis of the pooled data showed that the two groups did not differ significantly in the length of post-operative hospital stay (WMD = −0.05; 95% CI −0.18 to 0.08) (Figure 6).

### Meta-analysis of pathologic details

**Figure 7:**
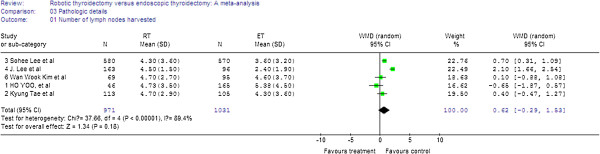
Forest plot displaying the results of the meta-analysis for number of lymph nodes harvested.

In the five studies, there was no significant difference between the two groups in the number of lymph nodes harvested (WMD = 0.62, 95% CI −0.29 to 1.53). The random-effects model was used because of the heterogeneity between the studies (I^2^ = 89.4%) (Figure 7).

## Discussion

Meta-analysis can be used for both qualitative and quantitative evaluation of existing literature by comparing and integrating the results of different studies and taking into account variations in characteristics that could influence the overall estimate of the outcome of interest [17]. Although meta-analysis has been traditionally applied and was mostly confined to randomized controlled trials (RCTs), meta-analytical techniques using nonrandomized controlled trials (NRCTs) might be a good method for use in some clinical settings in which either the number or the sample size of the RCTs is insufficient [18,19]. To our knowledge, this is the first comprehensive meta-analysis comparing RT versus ET.

RT is often perceived as being more time-consuming, because of the additional set-up time required [20]. Operating times depend mainly on the experience and skill of the surgeon. In this meta-analysis, we found that there was no significant difference in operating time between RT and ET. This may be attributable to the shortened learning curve with RT, as it has been suggested that robotic systems make the technique easier to learn, even by relatively inexperienced endoscopic surgeons [6]. With increasing experience, set-up time gradually decreased, and the actual time may be shorter in RT. There was no significant difference in conversion rates between RT and ET.

Although RT offers a number of advantages over ET, including improvements in manual dexterity, ergonomics, and visualization, the results of the present meta-analysis suggest that there is no additional clinical benefit for RT over ET. The disadvantages of RT are a higher rate of complications and a greater amount of drainage fluid. It has been suggested that the characteristics of RT might reduce complications because, using the Da Vinci Surgical System, robotic arms are used for retraction and dissection, and their use has been found to reduce unnecessary procedures and to minimize iatrogenic tissue injury during retraction. Consequently, our result is difficult to explain, and more studies are needed before such a conclusion can be drawn. There was no difference in post-operative hospital stay between the two groups, implying that the time required for patients to resume daily activities might be similar between RT and ET.

Oncologic outcomes after thyroid cancer surgery are affected by the extent of lymph-node dissection and the completeness of thyroidectomy [21,22]. Some studies have concluded that more lymph nodes are harvested via RT compared with ET, and that the robotic method may improve the long-term prognosis in patients who undergo surgery for thyroid cancer [13,14]. In this analysis, we found no significant differences between RT and ET in the number of lymph nodes harvested; however, long-term follow-up evaluation is necessary to evaluate the exact oncologic outcomes of RT for thyroid cancer.

In the studied articles we found significant heterogeneity in operating time and number of lymph nodes harvested, which may be explained by the differences in personnel skills, extension of lymph-node dissection, and period of the learning curve. Because of this heterogeneity, we used a random-effects model in this meta-analysis.

There are several limitations to this meta-analyis, and consequently, the results should be interpreted with caution. First, the data came from NRCTs, and the overall level of clinical evidence was low. It has been reported that NRCTs might either overestimate or underestimate the magnitude of the measured effect in an intervention study, regardless of quality scores [23]. However, Abrahama *et al*. found that meta-analyses carried out on well-designed NRCTs of surgical procedures were probably as accurate as those carried out on RCTs [24], and all six studies included in this study were NRCTs. Second, there was heterogeneity between the two groups because it was impossible to match the patient characteristics across all of the studies. We applied a random-effects model to take variation between studies into consideration, and we believe that the heterogeneity would have had very limited influence. Finally, it is possible that investigative groups might be more likely to report positive results, and that studies with significant outcomes are more likely to be published, therefore, potential publication bias might be present in our analysis.

## Conclusion

The results of this meta-analysis of 2,048 patients showed that RT was associated with an increased complication rate and a greater amount of drainage fluid after surgery, thus RT does not appear have any advantage over ET. Further studies are required to confirm these results.

## Competing interests

The authors declare that they have no competing interests.

## Authors’ contributions

YJR designed the study; CZH and YJR performed the literature search and retrieved data; LS and JHG collected the data; and LS and CZH performed the research and wrote the paper. All authors read and approved the final manuscript.
